# Genomic selection in timothy (*Phleum pratense* L.): a comprehensive evaluation of prediction models, multi-trait strategies, and forward validation across Norwegian environments

**DOI:** 10.1007/s00122-026-05371-x

**Published:** 2026-09-07

**Authors:** Mallikarjuna Rao Kovi, Akhil Reddy Pashapu, Helga Amdahl, Kristin Gylstrøm, Susanne Windju, Petter Marum, Muath Alsheikh, Odd Arne Rognli

**Affiliations:** 1 Department of Plant Sciences, Faculty of Biosciences, Norwegian University of Life Sciences (NMBU), Ås, Norway; 2 Graminor AS, Ridabu, Norway

## Abstract

**Key message:**

This study presents a comprehensive evaluation of genomic selection (GS) in timothy (*Phleum pratense* L.), comparing nine prediction models across yield and quality traits at two Norwegian locations. Forward validation with independent full-sib (FS2) families revealed a substantial generalization gap, highlighting the need for realistic accuracy assessment in polyploid forage breeding.

**Abstract:**

Timothy (*Phleum pratense* L.) is the most important forage grass in Northern Europe, yet genomic selection has not been systematically evaluated in this hexaploid species. We assessed 889 FS2-families originating from biparental crosses among 49 cultivars/populations. The FS2-families were genotyped with 30,698 SNP markers derived from genotyping-by-sequencing (GBS) and field tested for three harvest years at a highland and a lowland continental location in Southern Norway. Nine genomic prediction models were compared for six yield traits (dry matter yield per cut and total) and six quality traits (protein, digestibility, and fiber fractions) across three cuts/year. Within-training cross-validation accuracies were moderate to high (mean r = 0.62), with Random Forest and SVR consistently outperforming GBLUP. However, forward validation using 213 independent FS2-families revealed dramatically lower accuracies (mean r = 0.16), with only 16 of 30 trait–dataset combinations reaching statistical significance (*p* < 0.05). Genomic heritabilities (GREML), estimated across environments, ranged from near zero for the quality traits to 0.55 for the yield traits. Multi-trait models improved accuracy by 3–5% over single-trait approaches, while FS2 families-by-environment interaction models with Random Forest achieved the highest within-training accuracy (mean r = 0.71). Marker density analysis showed accuracy plateauing at approximately 15000 SNPs. Genetic correlations among the yield component traits were estimated by multi-trait REML; correlations among the quality traits could not be estimated reliably because their genomic heritabilities were low. A multi-trait selection index identified top-performing FS2-families for further crossing recommendations. These results provide a benchmark for GS implementation in hexaploid timothy and emphasize that cross-validation substantially overestimates prediction accuracy for truly independent material.

**Supplementary Information:**

The online version contains supplementary material available at https://doi.org/10.1007/s00122-026-05371-x.

## Introduction

Timothy (*Phleum pratense* L.) is the predominant cultivated forage grass species in the Nordic countries and one of the most important cool-season grasses worldwide (Berg et al. 1996; Casler 2012). In Norway, timothy constitutes approximately 70–80% of sown grasslands and is critical for livestock production systems at northern latitudes (Helgadottir et al. 2014). As an allohexaploid species (2n = 6x = 42) with a large and complex genome estimated at an approximate size of 4.25 Gb, timothy presents unique challenges for molecular breeding approaches that have been successfully applied in diploid grass species. Recent advances in genotyping technologies, particularly genotyping-by-sequencing (GBS), have made it feasible to generate genome-wide marker data in polyploid species, opening the door for genomic prediction approaches (Elshire et al. 2011; Poland and Rife 2012).

Genomic selection (GS), first proposed by Meuwissen et al. (2001), uses genome-wide marker information to predict breeding values of selection candidates without the need for direct phenotyping. This approach has revolutionized breeding programs in dairy cattle (Hayes et al. 2009; Wiggans et al. 2011) and is increasingly adopted in major crop species including wheat (Heffner et al. 2011; Crossa et al. 2014), maize (Bernardo and Yu 2007; Riedelsheimer et al. 2012), rice (Spindel et al. 2015), and barley (Lorenz et al. 2012). The core principle involves training statistical models on a reference population with both genotypic and phenotypic data, then applying these models to predict genomic estimated breeding values (GEBVs) for unphenotyped selection candidates (Jannink et al. 2010).

In forage grasses, GS has been explored primarily in perennial ryegrass (*Lolium perenne* L.), where prediction accuracies of 0.30–0.60 have been reported for yield and persistence traits (Pembleton et al. 2018; Arojju et al. 2020; Johansen et al. 2025). Studies in tall fescue (*Festuca arundinacea* Schreb.) and orchardgrass (*Dactylis glomerata* L.) have shown similar promise (Annicchiarico et al. 2015; Yamada et al. 2005). However, forage grass breeding programs face challenges that are particularly acute for GS implementation, including perenniality, extensive genotype-by-environment (G×E) interactions, and the importance of multiple traits including both yield and nutritive quality (Hayes et al. 2013; Lin et al. 2016; Johansen et al. 2025).

Despite the economic importance of timothy in Northern European agriculture, genomic selection has received limited attention in this species compared to other major crops. The hexaploid nature of timothy creates several methodological challenges: (1) complex patterns of allelic dosage that complicate SNP calling and interpretation (Clevenger et al. 2015; Bourke et al. 2018), (2) potential homoeologous recombination between subgenomes that affects linkage disequilibrium patterns (Mason and Wendel 2020), (3) large effective population sizes in outcrossing grass species that may reduce the extent of linkage disequilibrium (Ruttink et al. 2013), and (4) limited reference genome resources compared to diploid model species, although recent efforts have begun to address this gap (Shinozuka et al. 2012). These factors may affect the accuracy and transferability of genomic prediction models, making empirical evaluation essential.

Genomic prediction has begun to be explored in timothy specifically. In earlier work, we implemented genomic prediction in Norwegian timothy and examined the effect of genetic relatedness on prediction accuracy (Kovi et al. 2019; Rao Kovi et al. 2021), and, more recently, Vargas Jurado et al. (2025) evaluated genetic architecture and genomic prediction for yield, winter damage and digestibility in hexaploid timothy using genotyping-by-sequencing data, including analyses of marker density, single- and multiple-trait models, and forward prediction. The present study complements these works by comparing a broad set of nine prediction models and by contrasting within-training cross-validation with strict forward validation on an independently established set of FS2 families in Norwegian breeding germplasm.

A critical yet often overlooked aspect of genomic selection studies is the distinction between cross-validation (CV) accuracy and forward validation accuracy. Most GS studies in crops report CV estimates, where the training and testing sets are randomly sampled from the same population (Daetwyler et al. 2013). This approach can lead to optimistic accuracy estimates because related FS2 families may appear in both training and testing sets, inflating predictions through shared family structure rather than genuine marker-trait associations (Habier et al. 2010; Pszczola et al. 2012). Forward validation, using completely independent FS2 families not represented in the training population, provides a more realistic estimate of the accuracy achievable in practical breeding programs (Lorenz et al. 2011; Lozada et al. 2019). This distinction is particularly important in forage grass populations with strong family structure.

The choice of statistical model for genomic prediction is another important consideration. While linear mixed models such as GBLUP (genomic best linear unbiased prediction; VanRaden 2008; Endelman 2011) and Bayesian regression methods (Park and Casella 2008; Pérez and de los Campos 2014) are widely used, machine learning approaches including Random Forest (Breiman 2001; González-Recio and Forni 2011), Support Vector Regression (SVR; Vapnik 1995; Long et al. 2011), gradient boosting (Chen and Guestrin 2016; Ogutu et al. 2011), and neural networks (Montesinos-López et al. 2018; Ma et al. 2018) may capture non-additive genetic effects that are particularly relevant in polyploid species with complex epistatic interactions (Washburn et al. 2020). Furthermore, multi-trait models that exploit genetic correlations between traits (Calus and Veerkamp 2011; Jia and Jannink 2012), and genotype-by-environment (G×E) interaction models that account for differential genotype performance across environments (Burgueño et al. 2012; Jarquín et al. 2014; Lopez-Cruz et al. 2015; Johansen et al. 2025), may improve prediction accuracy over standard single-trait approaches.

The objectives of this study were to: (1) evaluate the accuracy of nine genomic prediction models in predicting yield and quality traits in full-sib families of timothy using 30,698 GBS-derived SNP markers; (2) compare within-training cross-validation with forward validation using a set of independent full-sib families; (3) assess multi-trait, multi-location, and G×E interaction models; (4) estimate genomic heritabilities and genetic correlations; (5) examine the effects of training population size and marker density on prediction accuracy; and (6) develop practical recommendations for GS-based FS2-family selection and crossing strategies in timothy breeding programs.

## Materials and methods

### Plant material and field trials

A total of 889 full-sib (FS2) families of timothy (*Phleum pratense* L.) originating from biparental crosses among 49 cultivars/populations were evaluated in field trials for 10 years from 2002–2012 at two contrasting locations in Southern Norway: Arneberg (60.7°N, 11.1°E; 180 m a.s.l.) in the continental inland region, and Løken (61.1°N, 9.1°E; 520 m a.s.l.) in the mountain region. Arneberg representing a warmer continental climate and Løken a cooler mountainous environment with a shorter growing season. The FS2-families were derived from controlled biparental crosses using cultivars and elite breeding populations including KGB, Grindstad, Liphlea, Motim, Carola, Richmond, Comer, Dolina, LøTi9941, and GnTi0301 as parents. At Arneberg and Løken, 613 and 823 FS2 families, respectively, were field tested with 612 FS2 families common to both locations. An additional independent validation set of 213 FS2 families was established in 2015 at both locations for forward validation of genomic predictions.

### Phenotypic evaluation

Yield traits were measured as dry matter (DM) yield (kg/ha) per cut for 3 years: KGDM101 (1st cut, year 1), KGDM102 (1st cut, year 2), KGDM201 (2nd cut, year 1), KGDM202 (2nd cut, year 2), KGDM301 (1st cut, year 3) and SUMDM (total DM yield summed across all cuts and years). Quality traits were assessed using near-infrared reflectance spectroscopy (NIRS) on samples from the 1 st, 2nd, and 3rd cuts. Six quality traits were predicted: crude protein content (Råprot, % of DM), acid detergent fiber (ADF, % of DM), neutral detergent fiber (NDF, % of DM), in vitro digestibility (Fordøy, % of DM), amino acid content available in the intestine (AAT, g/kg DM), and protein balance in the rumen (PBV, g/kg DM), following the Nordic AAT/PBV protein evaluation system (Madsen et al. 1995). Best linear unbiased predictions (BLUPs) were estimated for each FS2-family using mixed models fitted in ASReml (Gilmour et al. 2015) with FS2-family as random effect and year as fixed effect. A combined quality analysis was also performed by pooling observations from all three cuts. Descriptive statistics (number of records, mean, standard deviation, and range) for all yield and quality traits, by location and cut, are provided in Table S1.

### DNA extraction and genotyping-by-sequencing

Genomic DNA was extracted from pooled young leaf tissues of each family using a cetyltrimethylammonium bromide (CTAB) protocol adapted for grasses (Doyle and Doyle 1987). DNA concentration was quantified using a Qubit fluorometer (Thermo Fisher Scientific, Waltham, MA, USA), and quality was assessed by agarose gel electrophoresis. Genotyping-by-sequencing (GBS) was performed following the protocol of Elshire et al. (2011) with modifications for polyploid species. Briefly, genomic DNA was digested with the ApeKI restriction enzyme, ligated to barcoded adapters, and pooled for multiplex sequencing on the Illumina HiSeq platform (Illumina Inc., San Diego, CA, USA). Sequencing reads were demultiplexed and processed with the Stacks pipeline (Catchen et al. 2013) to build loci and call SNPs; the SNP set exported by the Stacks populations module was used for the quality-control filtering described below.

### SNP calling and quality control

Raw SNP calling yielded 60,635 biallelic SNPs across 915 FS2-family DNA pools. SNPs and FS2 families were subjected to stringent quality control filtering: (1) markers with minor allele frequency (MAF) < 0.05 were removed (655 markers); (2) families with > 40% missing marker data were excluded (26 FS2 families removed); and (3) markers with > 15% missing data across families were removed (29,282 markers). Following quality control, the final marker dataset comprised 30,698 polymorphic SNPs across 889 FS2-families. Missing genotype calls in the cleaned marker set were imputed using the Wright method as implemented in the snpReady R package (Granato et al. 2018). Given the hexaploid nature of timothy, markers were encoded using a diploidized biallelic representation, i.e., − 1, 0, 1 for homozygous reference, heterozygous, and homozygous alternative, respectively. In a hexaploid species, the alternative allele at a locus can be present in 0–6 copies. The allelic dosage was estimated for each locus using polyRAD (Clark et al. 2019) and then diploidized for the downstream analyses, so that the two homozygous dosage classes (0 and 6 copies) were coded − 1 and 1, respectively, and all heterozygous dosage classes (1–5 copies) were collapsed to a single heterozygous code 0. This has been shown to capture the majority of additive genetic variation in polyploid species (Endelman et al. 2018; Sverrisdottir et al. 2017). A Hardy–Weinberg equilibrium (HWE) filter was also applied prior to downstream analyses.

### Population structure analysis

Principal component analysis (PCA) was performed on the standardized marker matrix to characterize population structure and genetic relationships among the 889 FS2-families. Hierarchical clustering analysis using Ward’s method was applied to identify genetic subgroups at both locations. Phylogenetic dendrograms were constructed to visualize the genetic relationships among FS2-families and to assess the correspondence between molecular marker-based clusters and pedigree information.

### Experimental design for forward validation

To assess the realistic prediction accuracy achievable in a breeding program, we implemented a strict forward validation design following the recommendations of Lorenz et al. (2011) and Lozada et al. (2019). The dataset was partitioned into a training set (n = 413–505 FS2-families depending on trait and dataset) and an independent validation set of 213 FS2-families with zero overlap between sets. The 213 validation FS2-families were collected from an independent field evaluation cycle and were strictly excluded from the training population during model building. For yield data, the training set comprised 496 records at Arneberg and 723 at Løken after removal of validation FS2-families. For quality data, training sizes ranged from 488 to 505 depending on the cut. Within the training set, fivefold cross-validation was used to estimate within-training accuracy. The full training set was then used to build final models, which were applied to predict the independent validation FS2-families.

### Genomic prediction models

Nine genomic prediction models were evaluated, spanning classical linear mixed models, regularized regression, Bayesian methods, and machine learning approaches. Principal component analysis (PCA) was applied to the standardized marker matrix to reduce dimensionality prior to model fitting, retaining min (150, n−1) components for linear models and min (100, n−1) for machine learning models, where n is the training population size. This dimensionality reduction strategy balances information retention with computational efficiency and reduces the risk of overfitting. All 30,698 markers thus entered the analysis; no marker subset was used for the model comparison. The retained components captured approximately 25% (150 components, linear models) and 18% (100 components, machine learning models) of the total marker variance.

The nine models were: (1) GBLUP/Ridge regression, equivalent to GBLUP under the assumption of normally distributed marker effects (VanRaden 2008; Endelman 2011), implemented via Ridge regression with the regularization parameter selected by internal cross-validation; (2) LASSO (*α* = 0.01; Tibshirani 1996), which performs both variable selection and shrinkage; (3) Elastic Net (α = 0.01, l1_ratio = 0.5; Zou and Hastie 2005), combining L1 and L2 penalties; (4) Bayesian Ridge regression (Tipping 2001; Pérez and de los Campos 2014), with automatic determination of regularization parameters via evidence maximization; (5) Support Vector Regression (SVR; Vapnik 1995; Long et al. 2011), with RBF kernel and C = 1.0; (6) Random Forest (Breiman 2001; González-Recio and Forni 2011), with 200 trees and max_features = 0.33; (7) XGBoost gradient boosting (Chen and Guestrin 2016), with 150 trees and max_depth = 4; (8) LightGBM (Ke et al. 2017), with 150 trees and max_depth = 4; and (9) Multi-layer Perceptron (MLP) neural network (Montesinos-López et al. 2018), with two hidden layers (100 and 50 neurons) and ReLU activation. Regularization strengths noted above as selected by internal cross-validation (e.g., the Ridge/GBLUP penalty) were tuned by nested cross-validation within the training folds. The remaining hyperparameters (LASSO and Elastic-Net* α*, Elastic-Net l1_ratio, SVR C, Random Forest number of trees and max_features, the gradient-boosting number of trees and depth, and the neural-network architecture) were fixed a priori at the values given above, following common practice in the genomic-prediction literature, and were checked on a held-out development subset not to be sensitive within a reasonable range. All phenotypes were standardized (zero mean, unit variance) prior to model training.

The linear and Bayesian regressions share the base predictor y = 1μ + X*β* + e, where y is the vector of standardized phenotypes (assumed multivariate normal), X holds the retained principal-component scores, *β* are the marker-derived effects, and e are independent normal residuals. The methods differ in their assumptions about *β*: a Gaussian (ridge) prior for GBLUP/Ridge and Bayesian Ridge, an L1 penalty for LASSO, and a combined L1/L2 penalty for Elastic Net. The machine learning models relax the linearity and additivity assumptions and can capture non-additive effects.

### Multi-trait and multi-location models

Multi-trait (MT) models were implemented using MultiOutputRegressor wrappers (Pedregosa et al. 2011) for Ridge regression, SVR, and Bayesian Ridge, while Random Forest natively supports multi-output regression. MT models jointly predicted all yield or quality traits within a location; note, however, that the MultiOutputRegressor wrapper fits one independent model per trait, so for Ridge, SVR and Bayesian Ridge these predictions are mathematically equivalent to single-trait prediction and do not borrow strength across traits. Only Random Forest, through native multi-output regression, shares information across traits (Calus and Veerkamp 2011; Jia and Jannink 2012). Multi-location models were evaluated using three strategies: (1) pooled models that averaged phenotypes across locations for the 612 shared FS2-families (Burgueño et al. 2012); (2) genotype-by-environment (G×E) interaction models that included location as a binary covariate and marker-by-location interaction terms as additional features (Jarquín et al. 2014; Lopez-Cruz et al. 2015); and (3) combined multi-trait multi-location (MT-ML) models that simultaneously addressed both dimensions (approach (2) is a single-trait G×E reaction-norm model that adds location and marker-by-location terms; approach (3) additionally models the traits jointly). The G×E feature matrix was constructed by concatenating the original PCA marker scores, a location indicator, and element-wise products of marker scores with the location indicator.

### Genomic heritability estimation

Genomic heritability (h2_g_) was estimated by restricted maximum likelihood (GREML) as the proportion of phenotypic variance explained by a VanRaden genomic relationship matrix, with standard errors obtained by the delta method, following de los Campos et al. (2015). The same 30,698 markers were used as in the prediction models; because each FS2 family had a single record per environment, heritability was estimated across the two locations (yield) or three cuts (quality), which provide the replication needed to separate genomic from residual variance. Marker-based heritability was additionally estimated using restricted maximum likelihood (REML) methods as implemented in the heritability R package (Kruuk 2004; Piepho and Möhring 2007), using a genomic relationship matrix derived from the marker data.

### Marker density and training population size effects

The effect of marker density on prediction accuracy was assessed by randomly subsampling 5%, 10%, 20%, and 50% of the 30,698 markers (corresponding to approximately 1,534, 3,069, 6,139, and 15,349 SNPs) and comparing with the full marker set, following the approach of Heffner et al. (2011) and Lorenz et al. (2012). For each density level, three independent random subsamples were evaluated. Training population size effects were examined by using 20%, 30%, 40%, 50%, 60%, 70%, 80%, and 90% of the training data, with three replicates per fraction (Cericola et al. 2017; Norman et al. 2018). Both GBLUP and Random Forest were evaluated for these analyses. These two methods were chosen as representatives of the linear and nonparametric model families, which bracketed performance in the full comparison; the other linear methods behaved almost identically to GBLUP.

### Genetic correlations and confidence intervals

Genomic correlations between traits were estimated by multi-trait restricted maximum likelihood (REML) from a VanRaden genomic relationship matrix (variance and covariance components), using the two locations (yield) or three cuts (quality) as replication for identifiability, with approximate standard errors obtained by the delta method. Phenotypic correlations were computed from BLUP estimates for comparison. Bootstrap confidence intervals for prediction accuracies were calculated from 20 bootstrap replicates of the fivefold cross-validation procedure, reporting 2.5th and 97.5th percentile intervals (Efron and Tibshirani 1993).

### Selection index and crossing recommendations

A multi-trait selection index was constructed from clean GEBVs (estimated from training data excluding the validation set). The index is a weighted sum of standardized single-trait GEBVs from a single model (GBLUP/ridge regression) and does not use the estimated genetic-correlation matrix as an input, so the crossing recommendations are unaffected by the re-estimation of genetic correlations with trait weights reflecting breeding priorities for Norwegian forage production: SUMDM (3.0), Fordøy (2.0), KGDM101 (1.5), Råprot (1.0), PBV (0.5), NDF (− 1.0), and ADF (− 1.0). Each trait was standardized before weighting (Hazel 1943; Smith 1936). Crossing recommendations were generated for the top 30 FS2-families based on a crossing score combining normalized genetic merit (60% weight) and normalized genetic distance (40% weight), with a penalty factor of 0.7 for crosses within the same family to reduce inbreeding (Woolliams et al. 2015). Genetic distance was computed as Euclidean distance in 50-component PCA space (Jannink 2010).

### Statistical analysis and software

Genomic prediction analyses were implemented in Python 3 using scikit-learn v1.3 (Pedregosa et al. 2011) for model fitting and cross-validation, LightGBM v4.0 (Ke et al. 2017) and XGBoost v2.0 (Chen and Guestrin 2016) for gradient boosting methods, NumPy v1.24 (Harris et al. 2020) and pandas v2.0 (McKinney 2010) for data processing, and matplotlib v3.7 (Hunter 2007) for visualization. BLUPs and variance components were estimated using ASReml-R (Gilmour et al. 2015). Marker quality control and imputation were performed using the snpReady R package (Granato et al. 2018). Prediction accuracy was measured as the Pearson correlation (r) between predicted and observed values, and statistical significance of forward validation correlations was assessed using the cor.test function with a two-sided alternative hypothesis.

## Results

### Population structure and marker characteristics

After quality control filtering of the initial 60,635 GBS-derived SNPs, the final marker panel comprised 30,698 polymorphic SNPs across 889 FS2-families. PCA revealed weak population structure, with the first three principal components explaining only 0.6%, 0.4% and 0.4% of the total marker variance, respectively (Fig. S6), consistent with the very low relatedness among the FS2 families. Hierarchical clustering identified six major genetic subgroups at each location, broadly corresponding to the pedigree backgrounds of the full-sib (FS2) families. The KGB-derived families formed the largest cluster (346 FS2-families at Løken), consistent with the predominance of KGB breeding material in Norwegian timothy improvement. The 213 validation FS2-families were distributed across multiple clusters, though their representation was uneven across subgroups.

#### Genomic heritability

Genomic heritabilities, estimated by GREML from a genomic relationship matrix with standard errors (Table 1 and Fig. S1), were moderate for the yield traits. Because each FS2 family had a single field record per environment and the families are only distantly related, genomic and residual variances were not separately identifiable within a single location; heritabilities were therefore estimated across the two locations, which provide the replication needed for identifiability. Estimated genomic heritabilities were 0.50 ± 0.03 (KGDM101), 0.14 ± 0.04 (KGDM102), 0.43 ± 0.03 (KGDM201), 0.52 ± 0.03 (KGDM202), 0.55 ± 0.03 (KGDM301) and 0.37 ± 0.03 for total dry-matter yield (SUMDM).

**Table 1:** Genomic heritability (h2g) for yield and quality traits, estimated by GREML (restricted maximum likelihood) from a VanRaden genomic relationship matrix, with approximate standard errors (± SE)

Trait	Genomic h^2^g (GREML)	SE	Estimation basis
KGDM101	0.50	0.03	Yield (across locations)
KGDM102	0.14	0.04	Yield (across locations)
KGDM201	0.43	0.03	Yield (across locations)
KGDM202	0.52	0.03	Yield (across locations)
KGDM301	0.55	0.03	Yield (across locations)
SUMDM	0.37	0.03	Yield (across locations)
AAT	0.00	0.02	Quality (across cuts)
ADF	0.11	0.02	Quality (across cuts)
Fordøy	0.04	0.02	Quality (across cuts)
NDF	0.05	0.02	Quality (across cuts)
PBV	0.14	0.02	Quality (across cuts)
Råprot	0.08	0.02	Quality (across cuts)

Yield-trait heritabilities were estimated across the two locations (Arneberg and Løken) and quality-trait heritabilities across the three cuts, because each FS2 family had a single record per environment, which makes genomic and residual variances non-identifiable within a single environment

Genomic heritabilities for the quality traits, estimated across the three cuts, were low: 0.14 ± 0.02 (PBV), 0.11 ± 0.02 (ADF), 0.08 ± 0.02 (Råprot), 0.05 ± 0.02 (NDF), 0.04 ± 0.02 (Fordøy) and effectively zero for AAT (0.00 ± 0.02). These low values reflect both a modest additive genomic signal and substantial genotype-by-cut variation for quality traits, which were assessed at a single location, and are consistent with the generally weak forward validation obtained for quality traits.

#### Within-training cross-validation accuracy

Fivefold cross-validation (CV) within the training population revealed moderate to high prediction accuracies across traits and models. For yield traits at Arneberg, the best models achieved r = 0.77–0.82, with Random Forest and SVR consistently among the top performers (Table 2). SUMDM was the most predictable trait (RF: r = 0.82, 95% CI [0.82, 0.84]; SVR: r = 0.78 [0.77, 0.79]; GBLUP: r = 0.73 [0.70, 0.76]). At Løken, within-training accuracies were somewhat lower (best models: r = 0.42–0.71), reflecting the more challenging mountain environment and lower heritabilities.

**Table 2:** Summary of forward validation results for trait–dataset combinations with the best forward validation accuracy

Dataset	Trait	CV_r_	FV_r_	FV_p_	Significance	Best model	Gap
Arneberg	KGDM101	0.771	0.173	0.015	*	SVR	0.60
Arneberg	KGDM102	0.679	0.106	0.137		GBLUP	0.57
Arneberg	KGDM201	0.616	0.516	<0.001	***	SVR	0.10
Arneberg	KGDM202	0.705	0.209	0.003	**	SVR	0.50
Arneberg	KGDM301	0.678	0.050	0.486		RF	0.63
Arneberg	SUMDM	0.822	0.247	<0.001	***	GBLUP	0.58
Løken	KGDM101	0.615	0.311	<0.001	***	LightGBM	0.30
Løken	KGDM102	0.418	−0.059	0.403		Bay.Ridge	0.48
Løken	KGDM201	0.705	0.201	0.004	**	RF	0.50
Løken	KGDM202	0.575	0.089	0.213		XGBoost	0.49
Løken	KGDM301	0.674	0.055	0.436		LightGBM	0.62
Løken	SUMDM	0.651	−0.098	0.167		MLP	0.75
Q1	PBV	0.665	0.420	<0.001	***	SVR	0.24
Q1	Råprot	0.662	0.363	<0.001	***	LightGBM	0.30
Q1	NDF	0.545	0.220	0.002	**	XGBoost	0.32
Q2	Fordøy	0.593	0.190	0.007	**	SVR	0.40
Q2	PBV	0.595	0.216	0.002	**	RF	0.38
Q2	ADF	0.677	0.183	0.010	*	MLP	0.49
Q3	ADF	0.647	0.249	<0.001	***	RF	0.40
Q3	NDF	0.679	0.219	0.002	**	RF	0.46

CV_r_, best within-training cross-validation accuracy; FV_r_, best forward validation accuracy; FV_p_, *p* value of forward validation correlation

Significance: * *p* < 0.05, ** *p* < 0.01, *** *p* < 0.001. Gap = CV_r_—FV_r_

For quality traits, within-training CV accuracies ranged from r = 0.42 to 0.68 across cuts and traits. PBV and Råprot were the most predictable quality traits (r = 0.60–0.66), consistent with their higher genomic heritabilities. Machine learning models (RF, SVR, LightGBM) generally outperformed GBLUP by 5–15% for quality traits, while the advantage was smaller for yield traits (González-Recio and Forni 2011). The overall mean within-training CV accuracy across all 30 trait–dataset combinations was r = 0.62.

#### Forward validation accuracy

**Fig. 1:**
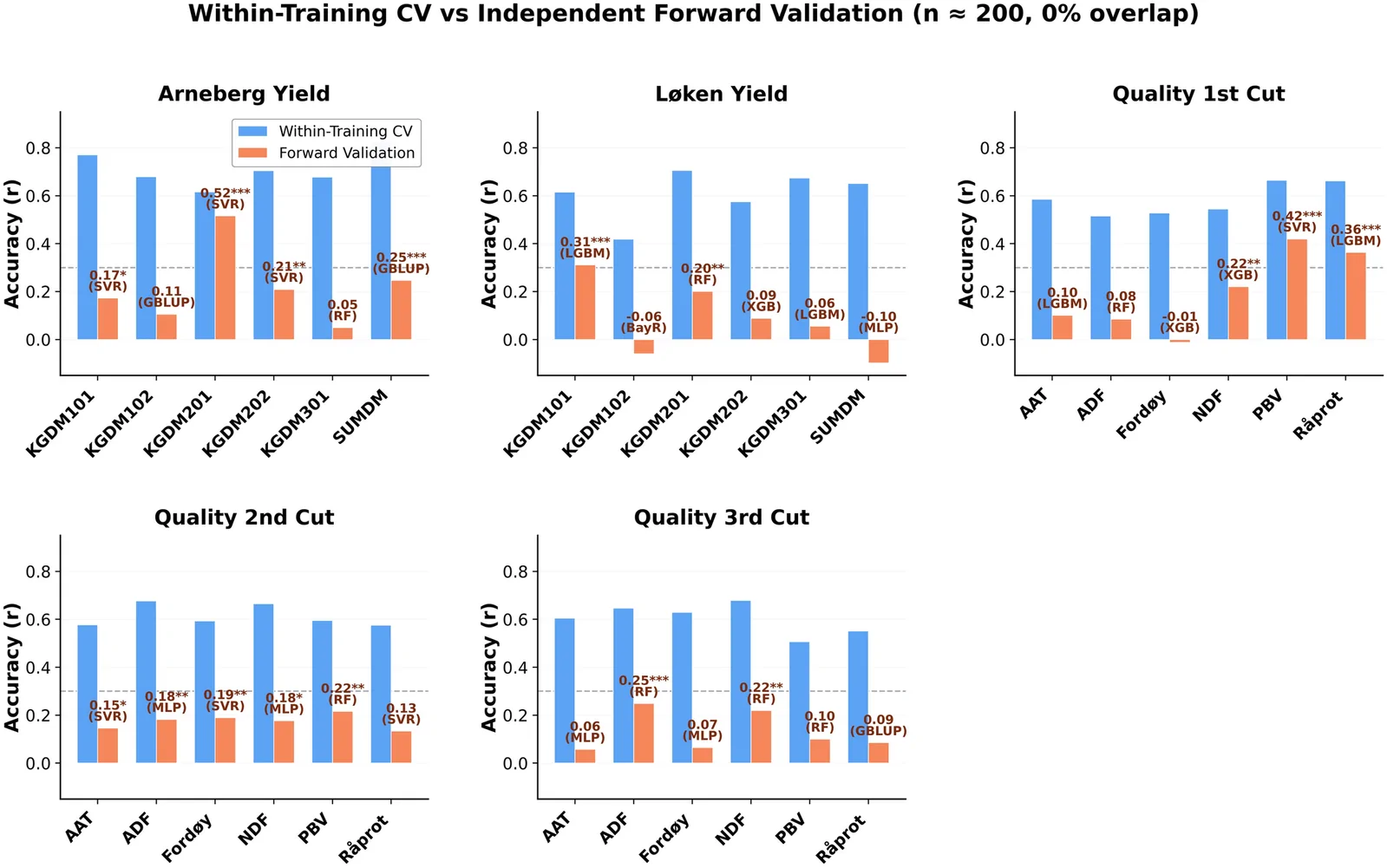
Within-training cross-validation accuracy versus independent forward validation accuracy for all trait–dataset combinations. Blue bars represent CV accuracy, and orange bars represent forward validation accuracy. The dashed gray line indicates the commonly used threshold (r = 0.3) for practical GS implementation. Asterisks denote statistical significance of forward validation correlation (p < 0.05)

**Fig. 2:**
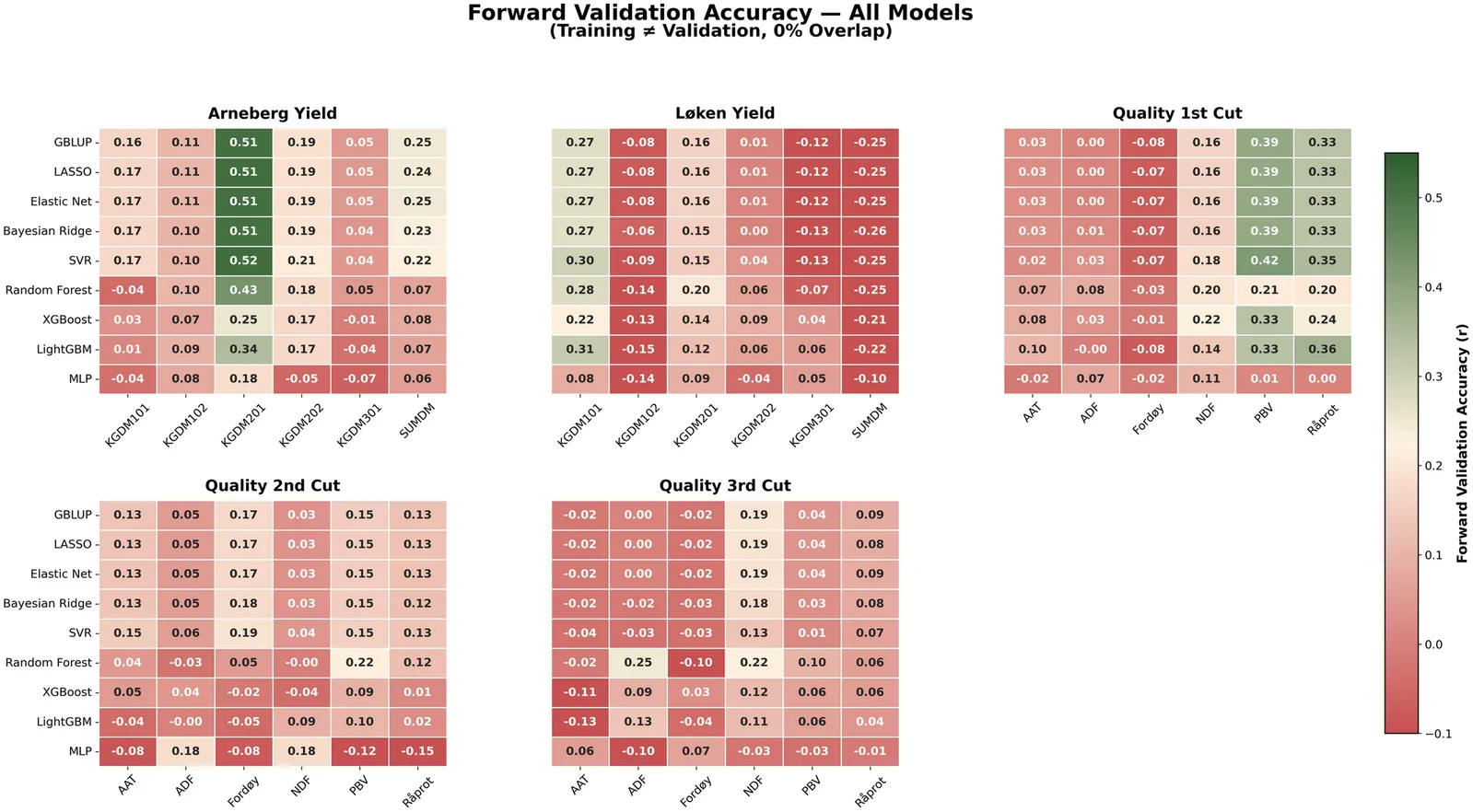
Forward validation accuracy heatmap across all nine prediction models and five datasets. Color scale ranges from red (negative or low accuracy) through yellow to green (high accuracy). Values represent Pearson correlations between predicted GEBVs and observed phenotypes in the independent validation set

Forward validation using the 213 independent FS2-families revealed substantially lower prediction accuracies compared to within-training CV (Fig. 1, Table 2). The mean forward validation accuracy across all 30 trait–dataset combinations was r = 0.16, representing a mean generalization gap of 0.46 from CV estimates. Only 16 of 30 combinations (53%) achieved statistical significance at *p* < 0.05 (Fig. 2).

**Fig. 3:**
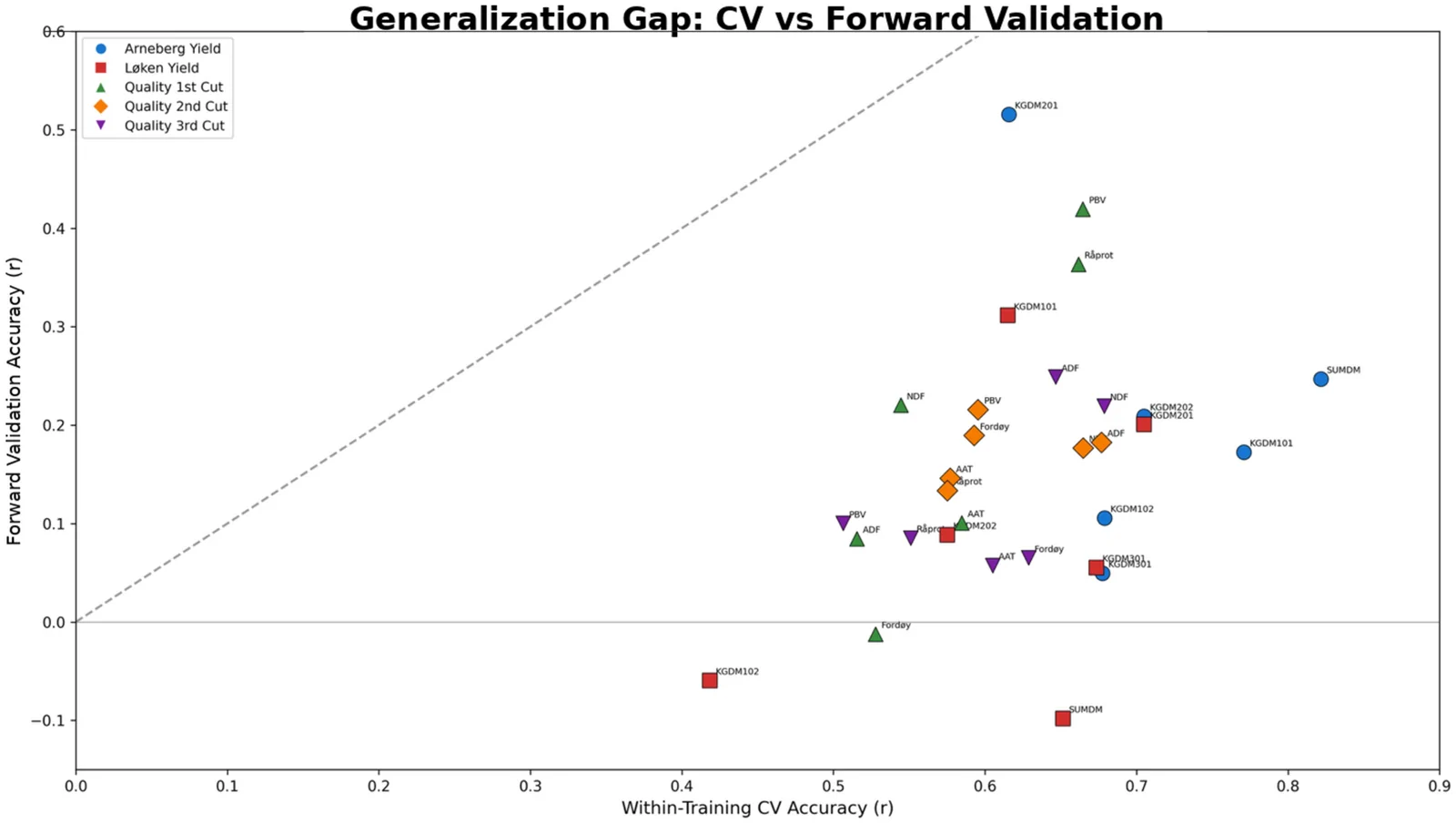
Generalization gap scatter plot. Each point represents one trait–dataset combination, with within-training CV accuracy (x-axis) and forward validation accuracy (y-axis). The dashed diagonal line represents y = x (no gap). Points below this line indicate accuracy loss in forward validation

For yield at Arneberg, the best forward validation results were obtained for KGDM201 (SVR, r = 0.52, *p* < 0.001) and SUMDM (GBLUP, r = 0.25, *p* < 0.001). At Løken, KGDM101 showed the best forward validation (LightGBM, r = 0.31, *p* < 0.001) and KGDM201 was also significant (RF, r = 0.20, *p* = 0.004). However, several traits showed non-significant or near or below-zero forward validation accuracy, particularly KGDM301 (r = 0.05 at Arneberg, *p* = 0.49) and KGDM102 at Løken (r =  − 0.06, *p* = 0.40) (Fig. 3).

Among quality traits, PBV consistently showed the highest forward validation accuracy across cuts (1st cut: SVR, r = 0.42, *p* < 0.001; 2nd cut: RF, r = 0.22, *p* = 0.002; 3rd cut: RF, r = 0.10, *p* = 0.16). Råprot also showed good forward validation in the 1 st cut (LightGBM, r = 0.36, *p* < 0.001). Fiber traits (ADF, NDF) showed moderate but significant forward validation in the 2nd and 3rd cuts (r = 0.18–0.25). The combined quality analysis (all three cuts pooled) showed significant forward validation for PBV (RF, r = 0.22, *p* = 0.002) and Råprot (RF, r = 0.21, *p* = 0.003), though with lower accuracy than individual-cut analyses.

Notably, the best model for forward validation often differed from the best cross-validation model, suggesting that models with highest within-training fit do not necessarily generalize best to independent material. GBLUP and SVR tended to show more robust generalization compared to complex ensemble methods, consistent with the regularization properties of these models (Daetwyler et al. 2013).

#### Model comparison across prediction methods

Among the nine models evaluated, machine learning methods generally achieved higher within-training CV accuracy but did not consistently outperform simpler methods in forward validation (Fig. S5). Random Forest showed the highest mean CV accuracy (r = 0.67), followed by SVR (r = 0.65) and XGBoost (r = 0.63). GBLUP achieved a mean CV accuracy of r = 0.58. However, in forward validation, SVR and GBLUP frequently achieved comparable or better results than ensemble methods. The neural network (MLP) showed high variance across trait–dataset combinations and occasionally achieved the best forward validation for individual traits but also produced negative correlations for some combinations. Bootstrap confidence intervals confirmed the superiority of RF and SVR within training (e.g., RF: 0.82–0.84 for Arneberg SUMDM) but also revealed wider intervals for complex models, indicating greater sensitivity to data composition.

#### Multi-trait and multi-location models

**Fig. 4:**
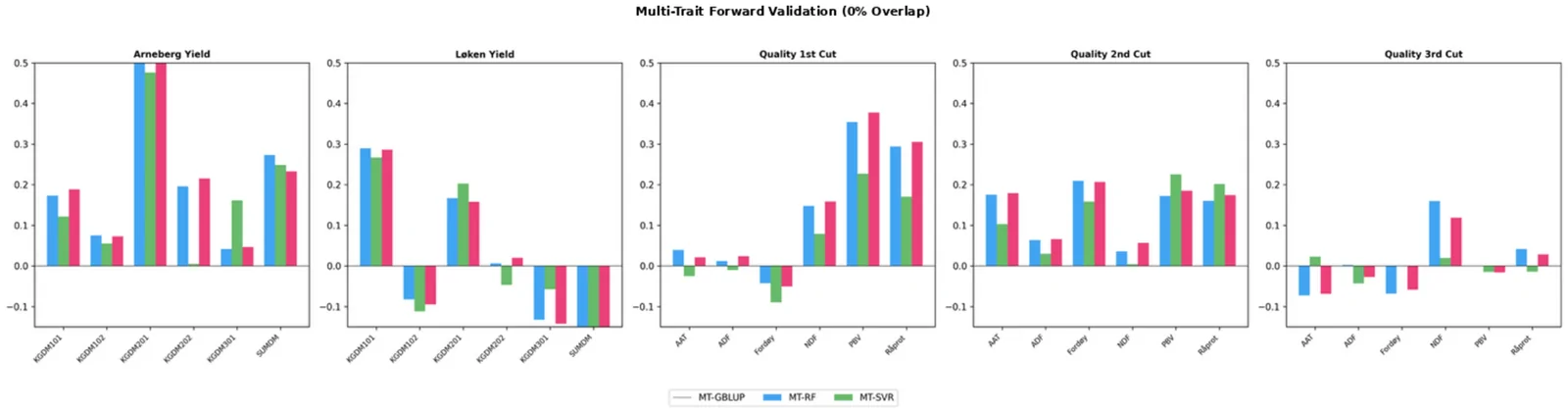
Multi-trait forward validation accuracy. Grouped bar charts comparing MT-GBLUP, MT-RF, and MT-SVR forward validation accuracy for each trait across five datasets

Multi-trait (MT) models provided modest but consistent improvements over single-trait models (Fig. 4). At Arneberg, MT-Random Forest achieved a mean within-training CV accuracy of r = 0.70 compared to r = 0.67 for the best single-trait model, a 3–5% improvement. At Løken, the improvement was similar (MT-RF: r = 0.63 vs single-trait RF: r = 0.60). For quality traits (1st cut), MT models achieved r = 0.51 (MT-RF), comparable to the best single-trait results. MT models were particularly beneficial for traits with low individual heritability, where information from correlated traits boosted prediction accuracy (Calus and Veerkamp 2011).

Multi-location models that pooled phenotypic data across Arneberg and Løken for the 612 shared FS2-families showed improved accuracy for some traits. The genotype-by-environment interaction (G×E) model, which included location as a covariate and marker-by-location interaction features, achieved the highest overall within-training accuracy (G×E-RF: mean r = 0.71), exceeding pooled models (Pooled-RF: r = 0.66) and single-location models. The G×E model showed particularly strong performance for KGDM202 (r = 0.74) and KGDM201 (r = 0.71). The combined multi-trait multi-location (MT-ML) model did not provide additional improvement over the G×E model alone (MT-ML-RF: mean r = 0.66), suggesting that the benefits of multi-trait and multi-location modeling are partially redundant when combined.

#### Marker density effects

Prediction accuracy increased with marker density but plateaued at approximately 15,000 markers (50% of the full panel) for both GBLUP and Random Forest (Fig. S3). For SUMDM at Arneberg, GBLUP accuracy increased from r = 0.54 with 1,534 markers (5%) to r = 0.73 with the full 30,698 markers, with the steepest gain occurring between 5 and 20% (1,534 to 6,139 markers). Random Forest showed a similar pattern, reaching r = 0.83 at 50% density with minimal improvement at 100%. For quality traits (Fordøy), GBLUP increased from r = 0.18 (5%) to r = 0.49 (100%), with the plateau less pronounced, indicating that quality traits may benefit from higher marker density.

#### Training population size effects

Prediction accuracy increased with training population size following a characteristic diminishing-returns curve (Fig. S2). For KGDM101 at Arneberg, GBLUP accuracy increased from r = 0.45 with 82 training FS2 families (20%) to r = 0.67 with the full 413 FS2-families. The rate of improvement decreased markedly above 60% (247 FS2-families), consistent with theoretical predictions that accuracy approaches an asymptote determined by the ratio of effective QTL number to population size (Daetwyler et al. 2008; Goddard 2009). Random Forest showed similar trends but with consistently higher absolute accuracies. Some instability was observed at very small training sizes (20–30%), likely due to insufficient sampling of the genetic diversity and family structure.

#### Genetic correlations

**Fig. 5:**
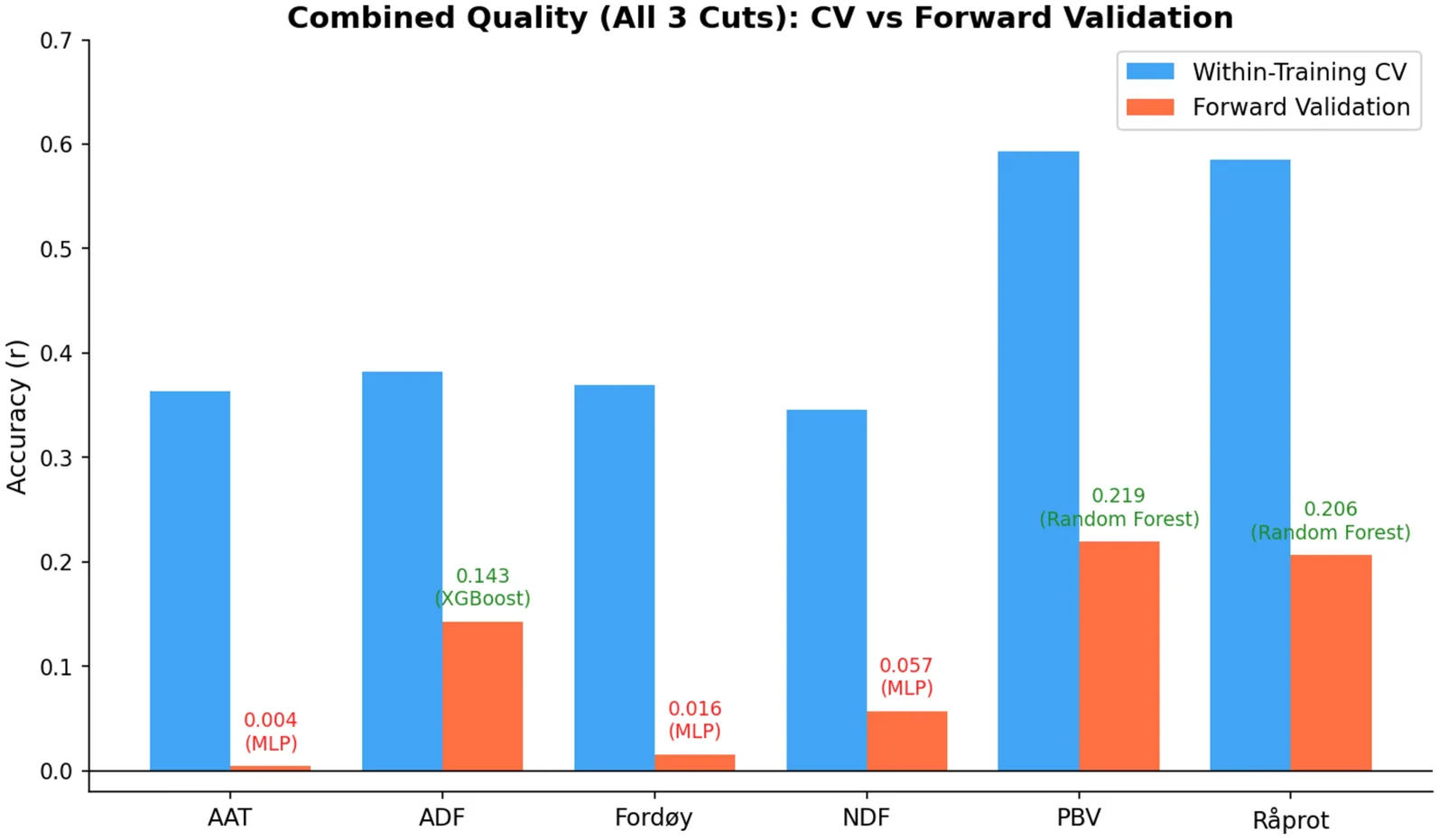
Combined quality trait validation: within-training CV versus forward validation for quality traits pooled across all three cuts. Abbreviations: Random Forest (RF), multi-layer perceptron neural network (MLP) and XGBoost gradient boosting (XGBoost)

Genetic correlations, estimated by multi-trait REML from the genomic relationship matrix (Fig. S4), were well estimated for the yield traits and are reported with approximate standard errors. Total dry-matter yield was strongly genetically correlated with its component cut yields, most strongly with the first cuts (r_g = 1.00 ± 0.24 with KGDM101, 0.76 ± 0.23 with KGDM201, 0.77 ± 0.19 with KGDM301), as expected given that SUMDM is their sum. First-cut and second-cut yields in year 1 were strongly correlated (KGDM101-KGDM201, r_g = 1.00 ± 0.20), whereas second-cut yield showed a weak-to-negative correlation between years (KGDM201-KGDM202, r_g = − 0.29 ± 0.08), indicating year-specific genetic control of regrowth (Fig. 5).

**Fig. 6:**
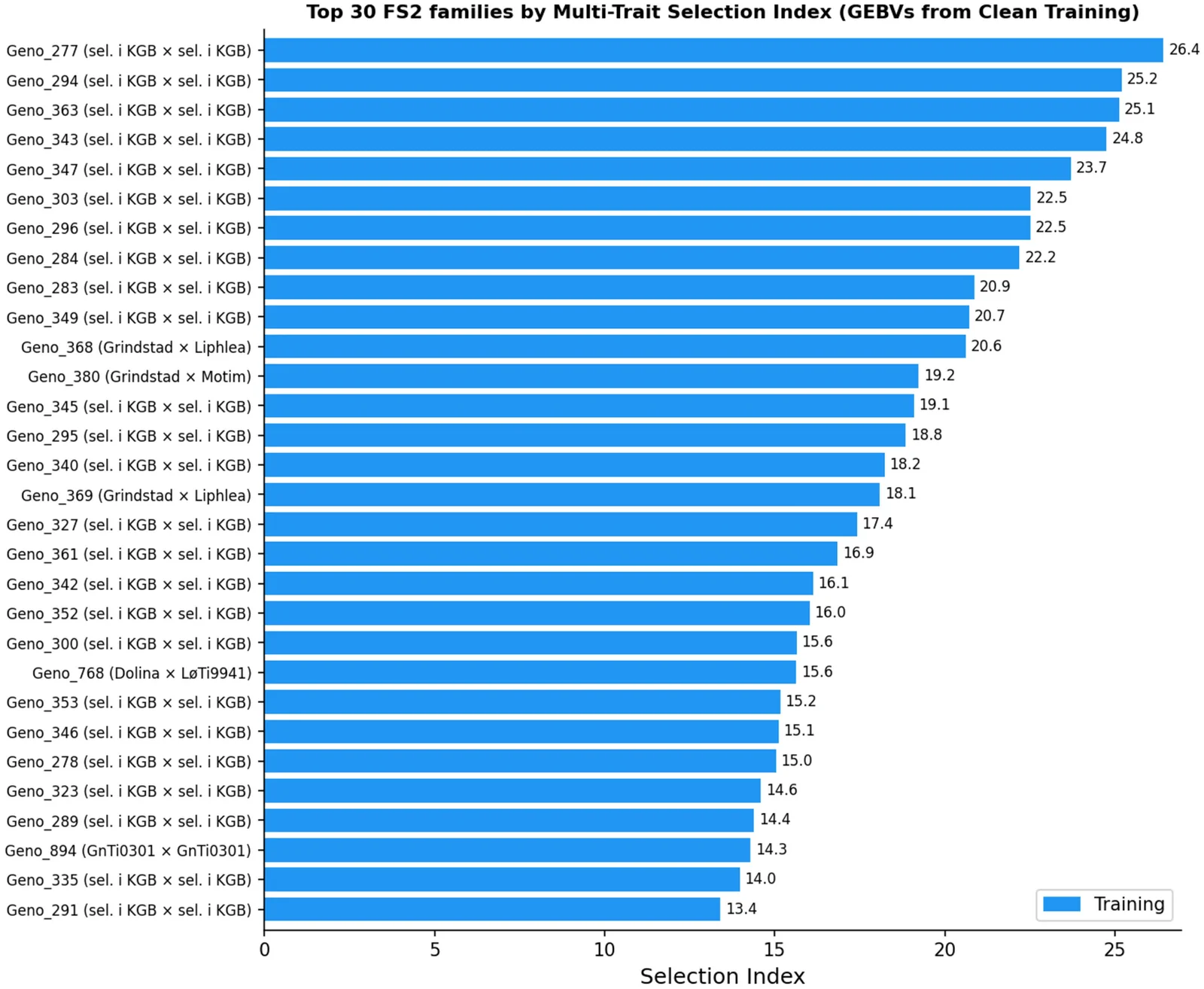
Top 30 FS2-families ranked by multi-trait selection index based on GEBVs from training models. All bars show training-set FS2 families. (There are no validation-set bars in this panel.) Labels include FS2-families ID and pedigree background

**Fig. 7:**
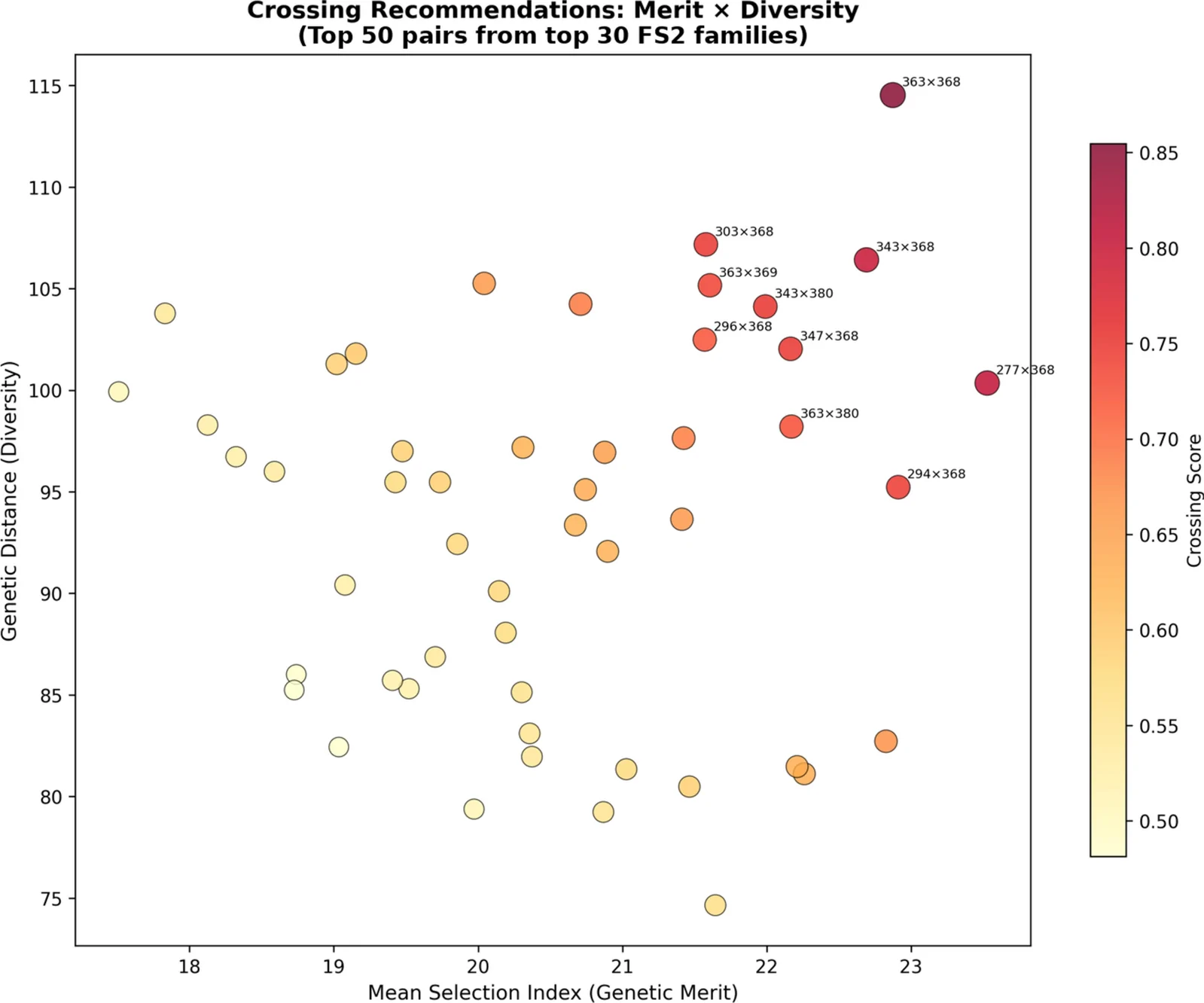
Crossing recommendations scatter plot: mean parental selection index (genetic merit) versus genetic distance (diversity) for top 50 candidate crosses. Color intensity represents crossing score (combining merit and diversity). Top 10 crosses are annotated

**Fig. 8:**
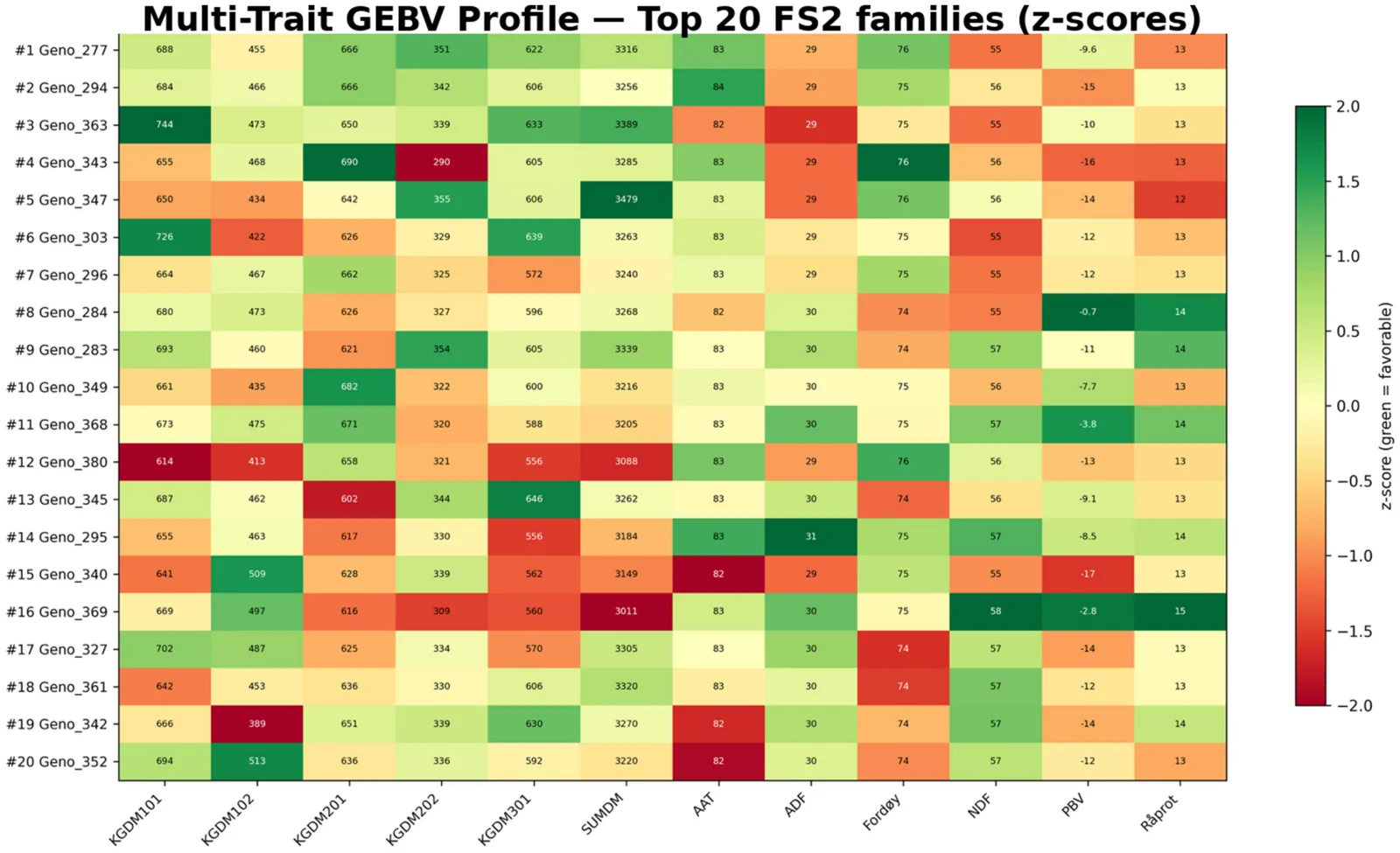
Multi-trait GEBV profile heatmap for the top 20 FS2-families ranked by selection index. Colors represent z-score standardized GEBVs (green = favorable, red = unfavorable)

For the quality traits, the low genomic heritabilities (Table 1) meant that the genomic (co)variances were too small to estimate most genetic correlations reliably. Where estimable, the fiber fractions ADF and NDF were strongly positively genetically correlated (r_g approximately 0.97), as expected because ADF is a structural component of NDF (Van Soest et al. 1991). We do not report precise genetic correlations for the protein traits (PBV, Råprot) or for AAT, because their genomic variances were too low to support reliable estimation; the near-perfect PBV-Råprot correlation reported previously was an artifact of correlating shrunken single-trait GEBVs (Figs. 6, 7, 8).

#### FS2-families ranking and selection recommendations

Based on GEBVs estimated from the training population, a multi-trait selection index identified the top 30 FS2-families for potential selection and crossing (Figs. 6 and 8). The top-ranked FS2-family was Geno_277 (Selection Index = 26.4), a progeny from a KGB × KGB cross, with high total DM yield (SUMDM = 3,316 kg/ha), good digestibility (Fordøy = 75.6%), and adequate crude protein (Råprot = 12.7%). The majority of top-ranked FS2-families (23/30) originated from KGB × KGB family crosses, with additional contributions from Grindstad × Liphlea, Grindstad × Motim, Dolina × LøTi9941, and GnTi0301 × GnTi0301 backgrounds.

Crossing recommendations for the top 30 FS2-families were optimized by balancing genetic merit with genetic distance (Fig. 7). The highest-scoring cross was Geno_363 × Geno_368 (crossing score = 0.85), combining a high-yielding KGB background with Grindstad × Liphlea parentage, maximizing both heterosis potential and genetic merit. Validation of GEBVs against actual phenotypes from the independent validation set confirmed the utility of GS-based selection: For the most predictable trait (KGDM201 at Arneberg), the GEBV-phenotype correlation was r = 0.51, and the top 20 FS2-families by GEBV had a mean phenotypic advantage of +107 kg DM/ha over the validation population mean.

## Discussion

### Comprehensive GS evaluation in hexaploid timothy

To our knowledge, this study represents a comprehensive evaluation of genomic selection in hexaploid timothy, complementing the recent evaluation of Vargas Jurado et al. (2025) and our earlier work (Rao Kovi et al. 2021); the present study is distinguished by its Norwegian breeding germplasm, the breadth of prediction models compared, and the use of strict forward validation on an independently established set of FS2 families (*Phleum pratense* L.), comparing a wide range of prediction models including both classical genomic prediction methods and modern machine learning approaches. Previous GS studies in forage grasses have focused primarily on perennial ryegrass (Pembleton et al. 2018; Arojju et al. 2020) and to a lesser extent tall fescue and orchardgrass (Annicchiarico et al. 2015; Yamada et al. 2005), which are diploid or lower-ploidy species. GS has also been evaluated in other polyploid crops including autotetraploid potato (Sverrisdottir et al. 2017; Endelman et al. 2018), allohexaploid wheat (Heffner et al. 2011; Crossa et al. 2014), and sugarcane (Gouy et al. 2013). The hexaploid nature of timothy, combined with its outcrossing mating system and large effective population size, creates a particularly challenging scenario for genomic prediction.

### The generalization gap: CV overestimates real-world accuracy

The most striking finding of this study is the large generalization gap between within-training cross-validation (mean r = 0.62) and forward validation accuracy (mean r = 0.16). This 74% reduction in accuracy when predicting truly independent FS2-families has profound implications for GS implementation in timothy breeding. While some generalization gap is expected and has been reported in other species, for instance, Lozada et al. (2019) reported 30–50% accuracy reductions in wheat, and Lorenz et al. (2012) observed similar patterns in barley, the magnitude observed here exceeds typical reports in diploid crops. The difference between cross-validation and forward-prediction accuracy reported for timothy by Vargas Jurado et al. (2025) was smaller than ours, most likely reflecting closer relatedness between their training and validation material; our validation FS2 families were established in an independent cycle and are only distantly related to the training families (mean genomic relationship near zero). Compression of the markers into a limited number of principal components (retaining roughly 18–25% of the marker variance) may also have contributed to the gap.

Several factors likely contribute to this large gap in timothy. First, the full-sib family structure of the population means that related FS2 families share both genetic background and long-range haplotype configurations. When related FS2-families appear in both CV training and testing folds, predictions may reflect family-level associations rather than marker-trait linkage, inflating accuracy (Habier et al. 2010; Pszczola et al. 2012). In forward validation, the 213 independent FS2-families may represent different genetic backgrounds, breaking these associations. Second, the hexaploid genome complexity means that diploidized markers may capture population-specific linkage patterns that do not transfer to independent material (Bourke et al. 2018). Third, environmental differences between the training and validation field trial cycles add noise to the forward validation signal. The observation that GBLUP and SVR generalized better than complex ML models is consistent with the bias-variance tradeoff: simpler models with stronger regularization trade some within-sample fit for better out-of-sample prediction (Daetwyler et al. 2013; Howard et al. 2014).

These results have important practical implications: GS breeding programs in timothy should be designed with realistic accuracy expectations based on forward validation rather than CV estimates. A forward validation accuracy of r = 0.20–0.50 for the most predictable traits (PBV, KGDM201, SUMDM) still represents meaningful genetic gain per unit time when combined with shortened generation intervals (Heffner et al. 2009; Bassi et al. 2016), as the response to selection is proportional to accuracy × selection intensity/generation interval (Falconer and Mackay 1996).

### Model performance and machine learning in polyploid GS

Within the training population, machine learning models (Random Forest, SVR, gradient boosting) consistently outperformed classical linear methods (GBLUP, LASSO, Bayesian Ridge) by 5–15%. This is consistent with the expectation that nonlinear models may better capture epistatic and dominance effects in polyploid species (Washburn et al. 2020; Montesinos-López et al. 2018). However, the advantage of complex models diminished in forward validation, where GBLUP and SVR often provided more robust predictions. This pattern has been observed in other species (Howard et al. 2014; Ogutu et al. 2012) and suggests that machine learning models may overfit to population-specific non-additive associations that do not transfer to independent material.

The G×E interaction model with Random Forest achieved the highest within-training accuracy (mean r = 0.71), demonstrating the value of explicitly modeling environment-specific effects in multi-location timothy trials. This 5% improvement over single-location models is consistent with G×E models in wheat (Burgueño et al. 2012; Lopez-Cruz et al. 2015) and suggests that G×E interactions are an important source of variation in timothy, reflecting the known sensitivity of this species to photoperiod, temperature, and precipitation regimes across Norwegian environments (Helgadottir et al. 2014).

### Marker density and training population considerations

The finding that prediction accuracy plateaus at approximately 15,000 markers has practical implications for genotyping strategies in timothy breeding programs. While the full 30,698 marker panel provides marginally higher accuracy, a cost-effective panel of 15,000–20,000 markers would achieve 90–95% of the maximum accuracy. This plateau is consistent with theoretical expectations based on the effective population size and extent of linkage disequilibrium in outcrossing polyploid species (Goddard 2009; Meuwissen et al. 2001). Targeted SNP panels or low-density arrays with imputation (Gorjanc et al. 2017) could further reduce genotyping costs while maintaining prediction accuracy.

Training population sizes of 250–300 FS2-families captured most available prediction accuracy, with diminishing returns beyond this threshold. This is consistent with simulation studies suggesting that optimal training population sizes depend on the ratio of effective QTL number to marker density and the level of linkage disequilibrium (Daetwyler et al. 2008; Lorenz et al. 2011). However, the forward validation results suggest that genetic diversity and representativeness of the training population may be more important than sheer size (Rincent et al. 2012; Isidro et al. 2015).

### Genetic correlations and multi-trait selection

The genomic heritabilities of the quality traits were low (Table 1), so their genetic correlations could not be estimated reliably in this material; the near-perfect PBV–Råprot correlation and the other strong quality-trait correlations reported in the initial analysis were artifacts of correlating shrunken single-trait GEBVs and are not retained. The generally expected antagonism between digestibility and fiber content (e.g., Fordøy and ADF) is consistent with forage-quality physiology (Van Soest et al. 1991) but would need to be quantified in larger, replicated trials. Among the yield traits, whose genomic heritabilities were moderate, multi-trait REML gave positive correlations of total DM yield with its component cuts, indicating that selection for total DM yield can be achieved through improvement of individual cut yields, although the weak correlation between consecutive second-cut yields (KGDM201 vs KGDM202, rg = − 0.29 ± 0.08) underlines the importance of multi-year evaluation.

The multi-trait selection index identified FS2-families from KGB backgrounds as consistently superior across yield and quality traits, reflecting the advanced breeding status of this population. The crossing recommendations, which balance genetic merit with genetic distance to maintain diversity and exploit heterosis (Woolliams et al. 2015; Jannink 2010), provide a practical framework for the next cycle of selection in Norwegian timothy improvement.

### Implications for timothy breeding programs

Despite the large generalization gap, these results support the potential utility of genomic selection in timothy breeding, particularly for traits with robust forward validation (PBV, Råprot, KGDM201, KGDM101). Even with forward validation accuracies of r = 0.20–0.50, GS can accelerate genetic gain by enabling early selection among unphenotyped seedlings and reducing the number of field evaluation cycles required before cultivar release (Heffner et al. 2009; Bassi et al. 2016; Lin et al. 2016).

Based on our findings, we recommend that timothy breeding programs: (1) establish genetically diverse training populations of at least 300 FS2-families, ensuring representation of the major breeding pools; (2) validate prediction models using truly independent FS2-families rather than relying solely on cross-validation; (3) employ 15,000–20,000 SNP markers as a cost-effective genotyping strategy, noting that GBS provides a practical platform for this scale; (4) interpret multi-trait models for the quality traits with caution, because the genomic heritabilities of the quality traits were low and their genetic correlations could not be estimated reliably in this material; (5) apply G×E models when multi-location data are available, as these provided the highest within-training accuracy; (6) use GBLUP or SVR as robust baseline models that generalize well to independent material; and (7) combine GS with phenotypic selection in a two-stage approach, where GS is used for initial screening and field evaluation is reserved for the top candidates.

Several limitations should be borne in mind. First, the hexaploid genome was represented by a diploidized (− 1/0/1) marker encoding, which ignores allelic dosage and may underestimate the available genomic information. Second, the markers were compressed into a limited number of principal components (retaining roughly 18–25% of the total marker variance), which may have reduced accuracy and contributed to the generalization gap; future work should evaluate models fitted directly to the full marker set. Third, the FS2 families are only distantly related and each was evaluated with essentially a single field record per environment, so genomic and residual variances were weakly identified within a single environment; genomic heritabilities (particularly for quality traits) and genetic correlations could therefore be estimated only across environments and, for low-heritability traits, only imprecisely. These features are characteristic of diverse, outcrossing breeding populations and reinforce the central message that forward validation, rather than cross-validation, should guide expectations for genomic selection in timothy.

## Conclusions

This study provides a comprehensive evaluation of genomic selection in hexaploid timothy grass, in Norwegian breeding germplasm, using 30,698 GBS-derived SNP markers across 889 FS2-families, revealing both the potential and limitations of GS in this important forage species. Within-training prediction accuracies were encouraging (mean r = 0.62), but forward validation with 213 independent FS2-families showed substantially reduced accuracy (mean r = 0.16), with only 53% of trait–dataset combinations reaching statistical significance. This generalization gap, larger than typically reported in diploid crops, underscores the importance of forward validation in polyploid GS studies. Machine learning models outperformed classical methods within training but showed less robust generalization. Multi-trait models improved accuracy by 3–5%, and G×E interaction models achieved the highest within-training accuracy (r = 0.71). A marker density of approximately 15,000 SNPs and training population of 250–300 FS2-families captured most available prediction information. These findings establish benchmarks for GS implementation in timothy and provide practical guidance for integrating genomic information into Northern European forage breeding programs.

## Supplementary Information

Below is the link to the electronic supplementary material.

Supplementary file1 (DOCX 725 kb)

Supplementary file2 (DOCX 11 kb)

## Data Availability

The cleaned marker matrix, and analysis scripts used in this study are stored at GitHub repository and available from the corresponding author upon request.
