# Supplementary material for: Genomic selection in timothy (Phleum pratense L.): a comprehensive evaluation of prediction models, multi-trait strategies, and forward validation across Norwegian environments
Source: Theor Appl Genet. 2026 Sep 7;139(10):262. doi: 10.1007/s00122-026-05371-x (PMC13550071; doi:10.1007/s00122-026-05371-x)
Supplement: Supplementary file 1 [file 122_2026_5371_MOESM1_ESM.docx]

**Fig. S1**. Genomic heritability (h²g) estimated by GREML (restricted maximum likelihood) for yield traits (across the two locations) and quality traits (across the three cuts). Error bars represent approximate standard errors

**Fig. S2.** Learning curves showing the effect of training population size (fraction of available training data) on GBLUP and Random Forest prediction accuracy for selected trait-dataset combinations.

**Fig. S3.** Effect of marker density on prediction accuracy for GBLUP and Random Forest. The x-axis shows the number of SNPs (log scale), and the y-axis shows mean cross-validation accuracy.

**Fig. S4**. Genomic (genetic) correlations among yield traits estimated by multi-trait REML across the two locations. Genetic correlations among the quality traits are not shown because their genomic heritabilities are too low for reliable multi-trait estimation (see Table 1).

**Fig. S5.** Model comparison with 95% bootstrap confidence intervals for selected trait-dataset combinations (Arneberg SUMDM, Løken SUMDM, Quality Fordøy). Error bars represent 2.5th-97.5th percentile intervals from 20 bootstrap replicates.

**Fig. S6**. Population structure of the 889 FS2 families. (a) Principal component analysis (PC1 vs PC2, coloured by genetic cluster) and (b) Ward hierarchical-clustering dendrogram, based on the 30,698 SNP markers. The first two components each explain well under 1% of the total marker variance, indicating weak population structure consistent with the low relatedness among the FS2 families.
