# Supplementary material for: Genomic selection in timothy (Phleum pratense L.): a comprehensive evaluation of prediction models, multi-trait strategies, and forward validation across Norwegian environments
Source: Theor Appl Genet. 2026 Sep 7;139(10):262. doi: 10.1007/s00122-026-05371-x (PMC13550071; doi:10.1007/s00122-026-05371-x)
Supplement: Supplementary file 2 [file 122_2026_5371_MOESM2_ESM.docx]

**Supplementary Material**

**Table S1.** Descriptive statistics of the phenotypic data used for genomic prediction: number of records (n), mean, standard deviation (SD), minimum and maximum for each yield and quality trait, summarised by location (yield traits) and by cut (quality traits). Trait abbreviations and units are as defined in the Materials and Methods (yield traits as dry-matter yield; quality traits from NIRS).

| **Dataset** | **Trait** | **n** | **Mean** | **SD** | **Min** | **Max** |
| --- | --- | --- | --- | --- | --- | --- |
| **Yield, Arneberg** | KGDM101 | 496 | 529.7 | 152.1 | 170 | 880 |
|  | KGDM102 | 496 | 432.2 | 134.8 | 191 | 745 |
|  | KGDM201 | 496 | 566.9 | 110.8 | 364 | 914 |
|  | KGDM202 | 496 | 274.4 | 122.7 | 77 | 576 |
|  | KGDM301 | 496 | 485.9 | 117.8 | 207 | 802 |
|  | SUMDM | 496 | 3002.3 | 448.2 | 1897 | 3763 |
| **Yield, Løken** | KGDM101 | 705 | 620.2 | 133.6 | 335 | 990 |
|  | KGDM102 | 687 | 428 | 132.2 | 185 | 858 |
|  | KGDM201 | 723 | 616.6 | 135.7 | 273 | 896 |
|  | KGDM202 | 723 | 375.8 | 101.7 | 177 | 718 |
|  | KGDM301 | 723 | 522.9 | 134.4 | 215 | 967 |
|  | SUMDM | 723 | 2811.0 | 494.9 | 1460 | 3899 |
| **Quality, 1st cut** | AAT | 590 | 77.47 | 2.65 | 70.30 | 84.90 |
|  | ADF | 590 | 33.88 | 1.58 | 29.70 | 38.30 |
|  | Fordøy | 590 | 69.85 | 2.21 | 63.60 | 75.80 |
|  | NDF | 590 | 63.51 | 2.70 | 55.30 | 70.60 |
|  | PBV | 590 | -22.24 | 22.27 | -85.90 | 17.00 |
|  | Råprot | 590 | 11.10 | 2.57 | 4.20 | 16.30 |
| **Quality, 2nd cut** | AAT | 573 | 79.67 | 3.89 | 70.80 | 89.10 |
|  | ADF | 573 | 32.27 | 2.00 | 26.00 | 36.30 |
|  | Fordøy | 573 | 72.48 | 3.69 | 63.30 | 80.80 |
|  | NDF | 573 | 59.77 | 3.27 | 48.50 | 66.50 |
|  | PBV | 573 | -26.47 | 24.77 | -76.50 | 31.60 |
|  | Råprot | 573 | 11.05 | 2.90 | 5.60 | 18.20 |
| **Quality, 3rd cut** | AAT | 576 | 85.90 | 2.54 | 79.00 | 91.60 |
|  | ADF | 576 | 26.04 | 2.17 | 21.40 | 31.60 |
|  | Fordøy | 576 | 77.25 | 2.74 | 71.30 | 83.20 |
|  | NDF | 576 | 50.45 | 3.34 | 42.30 | 61.00 |
|  | PBV | 576 | 13.60 | 18.16 | -24.70 | 51.90 |
|  | Råprot | 576 | 16.15 | 2.09 | 11.70 | 20.40 |

*Yield traits: KGDM101 to KGDM301 are per-cut dry-matter yields and SUMDM is total dry-matter yield summed across cuts and years. Quality traits: Råprot (crude protein), ADF (acid detergent fibre), NDF (neutral detergent fibre), Fordøy (in vitro digestibility), AAT (amino acids absorbed in the intestine) and PBV (protein balance in the rumen).*
